# RBC and WBC fatty acid composition following consumption of an omega 3 supplement: Lessons for future clinical trials

**DOI:** 10.1186/1476-511X-9-31

**Published:** 2010-03-22

**Authors:** Theodore R Witte, Alexander J Salazar, Oscar F Ballester, W Elaine Hardman

**Affiliations:** 1 Department of Biochemistry and Microbiology, Marshall University School of Medicine, Huntington, West Virginia, USA; 2 Edwards Comprehensive Cancer Center, Marshall University School of Medicine, Huntington, West Virginia, USA

## Abstract

**Background:**

Results from increasing numbers of *in vitro *and *in vivo *studies have demonstrated that omega 3 fatty acids incorporated in cell culture media or in the diet of the animals can suppress the growth of cancers. When human clinical trials are initiated to determine the ability of omega 3 fatty acids to alter growth or response to chemotherapeutic interventions of cancers, it will be essential to determine the omega 3 intake of individuals in the trial to determine compliance with consumption of the supplement and to correlate with endpoints of efficacy. We wondered if the fatty acid composition of RBCs might accurately indicate incorporation of omega 3 fatty acids in the WBCs. In this report we determine and compare the changes in fatty acid compositions of red blood cells and white blood cells in response to consumption of three doses of an omega 3 fatty acid supplement.

**Results:**

We found that the fraction of omega 3 fatty acids in both red blood cells and white blood cells increased following consumption of the supplement. There was a linear, dose responsive increase in the fraction of omega 3 fatty acids in red blood cells but the increase in omega 3 in white blood cells was not linear. The magnitude of increase in omega 3 fatty acids was different between the two cell types.

**Conclusions:**

Fatty acid analysis of red blood cells is a good measure of compliance with supplement consumption. However, fatty acid analysis of white blood cells is needed to correlate changes in fatty acid composition of white blood cells with other biochemical changes in the white blood cells.

**Trial Registration:**

ClinicalTrials.gov NCT00899353.

## Introduction

Humans are unable to synthesize the omega 3 or omega 6 bonds, thus, omega-3 and omega-6 fatty acids are essential fats which we must obtain from food. The ratio of omega-3 to omega-6 fats in the average western and/or modern diet is heavily weighted in favor of omega-6[1]. Omega-3 deficiencies have been implicated in growth retardation, neurological dysfunction, and excessive blood clotting[1]. In recent years, results from increasing numbers of *in vitro *and *in vivo *studies have demonstrated that omega 3 fatty acids incorporated in cell culture media or in the diet of the animals can suppress the growth of cancers [summarized in [2]]. When human clinical trials to determine the ability of omega 3 fatty acids to alter growth or response to chemotherapeutic interventions of cancers are initiated, it will be essential to determine the omega 3 intake of individuals in the trial to correlate with endpoints of efficacy. It will also be useful to determine the omega 3 incorporation in specific tissues to correlate change in fatty acid composition of the tissue with response to therapy.

A commonly used measure of intake and incorporation of dietary or supplementary fatty acids into cells is the gas chromatographic analysis of lipid composition of the specimen[3,4]. In future clinical trials, specific tissues or cells of interest may be difficult or highly invasive to obtain or of limited availability, however, procuring blood specimens is a relatively non-invasive procedure. Assessing the composition of red or white blood cells has been used as a marker of omega 3 fatty acid intake in previous reports[3,4]. In our animal studies, we have reported fatty acid composition of tissues as a marker for dietary consumption of omega 3 fatty acid [5-7]. It should be noted that change in fatty acid composition due to diet was different for different tissues, i.e. the composition of tumor, liver and fat all changed in response to diet but the magnitude of change was unique to each tissue[7].

We are performing pilot studies for a human clinical trial. In this trial, patients with early stage chronic lymphocytic leukemia (esCLL) received an omega 3 fatty acid supplement in increasing doses with the immediate goal of reducing expression of nuclear factor kappa B. Our long term goal (which cannot and will not be assessed in this pilot study) is delaying progression of disease or increasing the response of the patients to chemotherapy when they do progress to the point of needing chemotherapy. The white blood cells (WBCs) of these patients are far fewer in the blood than the red blood cells (RBCs), thus we wondered if the fatty acid composition of RBCs might accurately indicate incorporation of omega 3 fatty acids in the WBCs. In this report, we address the: 1) rate of change in the omega 3 composition of RBCs and WBCs following omega 3 fatty acid consumption and 2) comparability of RBC and WBC composition before and after supplementation. Previous papers have shown that the fatty acid compositions of both RBCs[3,8] and WBCs[9,10] change in response to omega 3 fatty acid consumption but to our knowledge there are not reports that compare baseline RBCs and WBCs fatty acid compositions or compare the change in RBCs fatty acid composition to the change in WBCs fatty acid composition to assess the value of using change in RBC fatty acid composition as a surrogate for change in WBC fatty acid composition in future clinical trials.

## Results

### Baseline fatty acid composition of red and white blood cells

**Figure 1:**
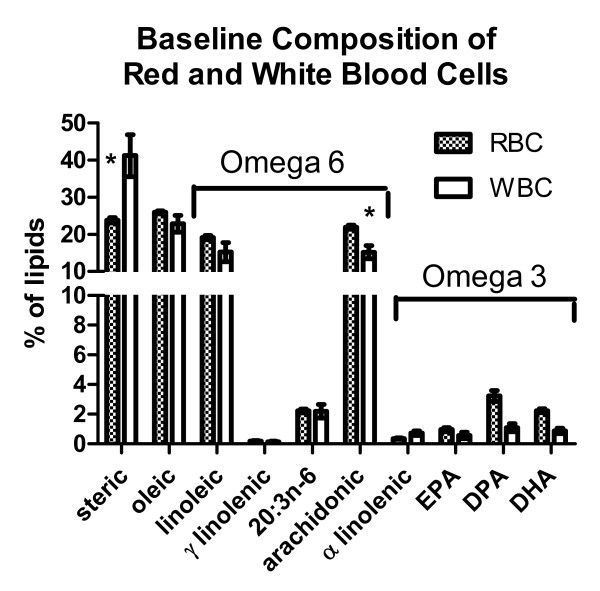
**Baseline composition of red and white blood cells**. The % compositions (mean ± SEM) of ten assayed lipids at baseline (without omega 3 supplement) of red and white blood cells are shown. Two way analyses of variance followed by Bonferroni posttests showed that the steric acid fraction was significantly less and the arachidonic acid fraction was significantly higher in red blood cells than in white blood cells at baseline. EPA - eicosapentaenoic acid; DPA - docosapentaenoic acid; DHA - docosahexaenoic acid.

The fatty acid compositions of red and white blood cells of patients at baseline are compared in Figure 1. Two way analyses of variance followed by Bonferroni posttests showed that the steric acid fraction was significantly less and the arachidonic acid fraction was significantly higher in RBCs than in WBCs. There were no other significant differences.

### Red blood cells response to omega 3 supplement

**Figure 2:**
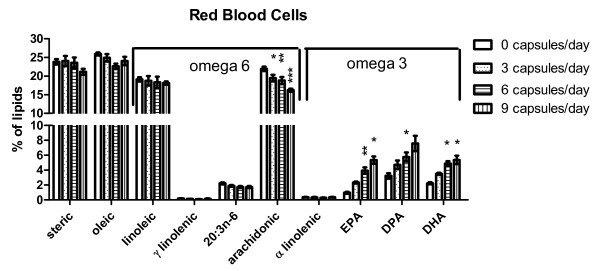
**Lipid composition of red blood cells**. The % compositions (mean ± SEM) of ten assayed lipids are shown. Omega 6 and omega 3 species are identified by the bracket over the top of each set. Repeated measures analyses of variance followed by Bonferroni posttests were used to determine significant changes from the baseline fraction of each fatty acid. Significant differences from baseline following each dose of omega 3 supplement are shown by: * - p < 0.05; ** p < 0.01; *** - p < 0.001. EPA - eicosapentaenoic acid; DPA - docosapentaenoic acid; DHA - docosahexaenoic acid.

The fatty acid compositions of RBCs of patients at baseline and after consuming 3, 6 or 9 capsules of omega 3 supplement for one month are shown in Figure 2. As expected, linear regression analyses showed there were significant dose responsive decreases in the omega 6 fatty acids: (slope mean ± SE % per capsule, p value for slope of line is significantly different from a slope of 0, r^2 ^= correlation coefficient) linoleic acid (slope = -0.1124 ± 0.01257, p = 0.01, r^2 ^= 0.97) and arachidonic acid (slope = -0.5956 ± 0.08963, p = 0.02, r^2 ^= 0.96). There were significant, dose responsive increases in the omega 3 fatty acids: EPA (slope 0.4924 ± 0.01134, p = 0.0005, r^2 ^= 0.99), DPA (slope 0.4690 ± 0.03361, p = 0.005, r^2 ^= 0.99) and DHA (slope 0.3593 ± 0.04765, p = 0.017, r^2 ^= 0.97). There were no other significant linear regressions for the other measured fatty acids.

Repeated measures two way analyses of variance was used to determine significant differences in the individual omega 6 and omega 3 fatty acids of RBCs between baseline and after consumption of 3, 6 or 9 capsules of supplement for one month to determine the minimum dose required for a mathematically significant change. The fraction of arachidonic acid in RBCs was significantly less than baseline after consumption of 3 or more (p < 0.05) capsules per day. The fractions of EPA (p < 0.01), DPA (p < 0.05) and DHA (p < 0.05) in RBCs were significantly more than baseline after consumption of 6 or more capsules per day for one month.

### White blood cells response to omega 3 supplement

**Figure 3:**
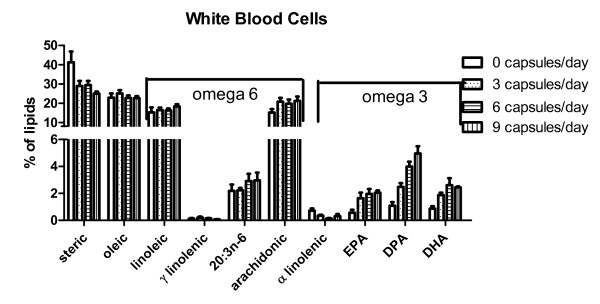
**Lipid composition of white blood cells**. The % compositions (mean ± SEM) of ten assayed lipids are shown. Omega 6 and omega 3 species are identified by the bracket over the top of each set. Repeated measures analyses of variance followed by Bonferroni posttests showed that there were no significant changes from the baseline fraction of individual fatty acids. EPA - eicosapentaenoic acid; DPA - docosapentaenoic acid; DHA - docosahexaenoic acid.

The fatty acid compositions of WBCs from patients at baseline and after consuming 3, 6 or 9 capsules of omega 3 supplement for one month are shown in Figure 3. The change in fatty acid composition of the WBCs in response to the omega 3 supplement was quite different from that seen for the RBCs. Linear regression analyses showed that the only significant dose response was that of DPA (slope 0.4377 ± 0.02766, p = 0.004, r^2 ^= .99). The incorporation of both EPA (slope 0.1594 ± 0.05334, p = 0.1, r^2 ^= 0.82) and DHA (slope 0.1821 ± 0.06349, p = 0.1, r^2 ^= 0.80) were not quite dose responsive, probably because increasing the dose from 6 to 9 capsules did not increase the amount of EPA and DHA in cell membranes.

Repeated measures two way analyses of variance was used to determine significant differences in the individual omega 6 and omega 3 fatty acids of WBCs between baseline and after consumption of 3, 6 or 9 capsules of supplement for one month. There were no mathematically significant changes in omega 6 or omega 3 fatty acids in WBCs membranes.

### Change in sum of omega 3 fatty acids for RBCs and WBCs

**Figure 4:**
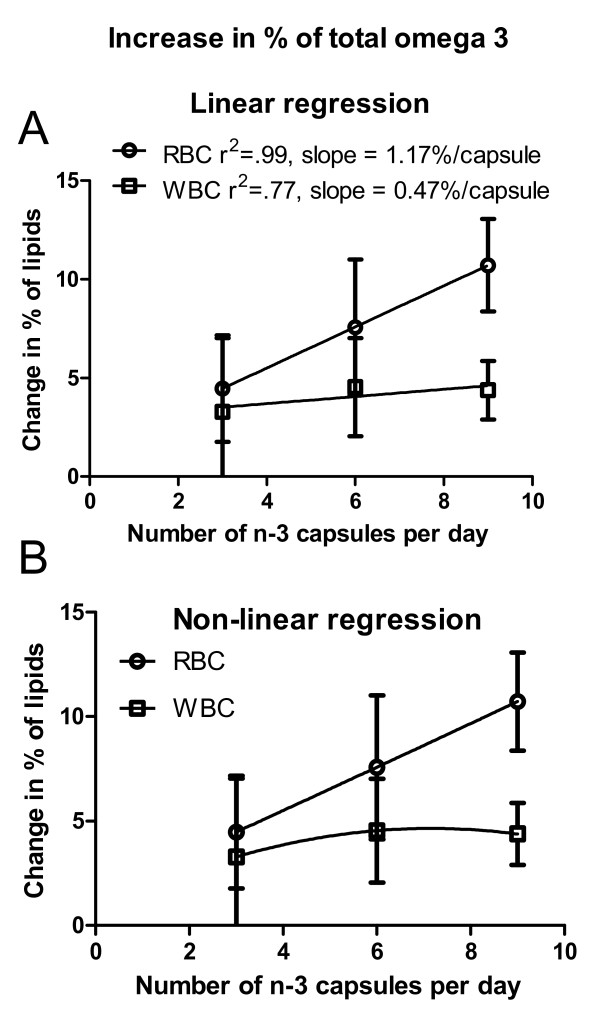
**Change in total omega 3 in red and white blood cells**. The change in the sum of % compositions of omega 3 fatty acids in red or in white blood cells following consumption of 3, 6 or 9 capsules of omega 3 supplement for one month is shown. A. Linear regression was used to determine the dose responsive change in omega 3 fatty acids in red or white blood cell membranes. For red blood cells, there was a significant linear regression, r^2 ^= 0.99, slope = 1.17%/capsule. The regression fit was not significant for white blood cells. The correlation coefficient for a best fit line was 0.77 with a slope of 0.47%/capsule. B. Non-linear regression was a better fit to the change in lipid composition of white blood cells.

Figure 4 illustrates the increases from baseline in the total fraction of omega 3 fatty acids (sum of ALA, EPA, DPA and DHA) in membranes of red and white blood cells after consumption of 3, 6 or 9 capsules per day for one month. Three points are clear from these graphs: 1) RBCs incorporate a higher fraction of omega 3 fatty acids than WBCs; 2) Figure 4A shows that the incorporation of omega 3 in RBCs was linear (slope = 1.17%/capsule, r^2 ^= .99) and dose responsive; 3) The incorporation of omega 3 in WBCs was not linear and was at a slower rate than RBCs. The best linear fit for incorporation of omega 3 in WBCs had a slope of 0.47%/capsule (R^2 ^= 0.77), less than 1/2 the slope for incorporation of omega 3 in RBCs. As shown in Figure 4-B, a non linear fit was a better fit for the incorporation of omega 3 fatty acids in WBCs. Two way ANOVA indicated a significant difference due to cell type (p = 0.0078), that is, the omega 3 fraction in WBCs was always less than the omega 3 fraction in RBCs.

## Discussion

It is important to have some measure of compliance in clinical trials, whether the test substance is a drug or consumption of a dietary supplement. Results in previous papers have demonstrated that the fatty acid composition of both RBCs [3,8] and WBCs [9,10] does change in response to supplementation of the diet with omega 3 fatty acids. It has been reported that the composition of RBCs is less variable and thus a better marker for longer term consumption than plasma[11]. However, in designing the clinical trial for which these specimens were obtained we could not find comparisons of changes in WBC and RBC fatty acid compositions in response to an omega 3 supplement. It was important to us to know that the omega 3 supplement was being incorporated into RBCs and into WBCs so that we could assess compliance with supplement consumption in our patients and so that we could later relate the changes in fatty acid composition in the tumor cells (i.e., early stage chronic lymphocytic leukemia) with other measured parameters.

The results reported herein indicate that the question determines whether the fatty acid composition of RBCs or WBCs is the best marker. If the question is about patient compliance with the consumption of the omega 3 supplement, then the fatty acid composition of RBCs is clearly a good marker. The increase of total omega 3 content in the RBCs was linearly related to consumption of the omega 3 supplement. We stressed to the patients that if they could not take an increased dose of the supplement, that was fine, but we did need to know the actual consumption of capsules. Our patients reported compliance with the prescribed dose of supplement or reported the actual reduction in consumption. Pill counts supported those reports.

On the other hand, the fatty acid composition of the WBCs, obtained from the same blood specimen as the RBCs did not show a dose responsive increase in omega 3 fatty acids. The fraction of omega 3 fatty acids showed the biggest increase from baseline to 3 capsules per day, an increase of 3.2%. The increase from 3 to 6 capsules was less than half as much, an additional 1.3% to a total of 4.5% of the total fatty acids were omega 3. There was no additional increase when the consumption of the omega 3 supplement was increased to 9 capsules per day. Clearly the fatty acid composition of the WBCs would not be a good indicator of compliance with consumption of the supplement in future clinical trials.

These results may also help us determine dosage for future clinical trials in which omega 3 fatty acid supplementation is tried as an adjuvant for chemotherapy. Animal studies indicate that supplementation of the diet with omega 3 fatty acids increases the efficacy of chemo and radiation therapy [12-21]. Incorporation of the omega 3 fatty acids into cell membranes is required for the activity of the omega 3. Usually only one dose of omega 3 was incorporated in the diet for animal studies rather than a range of doses to determine if there was a dose response. The drug paradigm for chemotherapy is to use the largest dose the patient can tolerate without detrimental side effects. However, the results shown here indicate that 9 capsules per day did not result in more omega 3 in WBCs than did 6 capsules per day for one month. We do not know if more than 9 capsules per day would result in another increase in omega 3 in the WBCs. The reason the number of patients at 9 capsules per day is small is that, even though there were few reported side effects, few patients were willing to take 9 capsules per day. If patients will not take the supplement if does not matter if more would have been better. Our results indicate that the fraction of omega 3 fatty acids in WBC cell membranes was the same after 6 capsules of this supplement as after 9 capsules of the supplement.

Several questions are still unanswered: 1)What is the rate of disappearance of omega 3 after discontinuing the omega 3 supplement? 2) Once a high level of omega 3 is reached in the WBCs membranes, can this level be maintained with a smaller dose of supplement? These questions will be important to answer for future clinical trials with the least burden to the patients.

## Conclusions

In conclusion, at baseline, there was little difference between red blood cells and white blood cells in fatty acid composition. The fatty acids composition of RBCs is a dose responsive indicator of omega 3 consumption but the fatty acid composition of WBCs does not change linearly with dose. Because of the differences between RBCs and WBCs in dose response, the composition of the WBCs must be assessed if one wants to relate further biochemical changes to the amount of omega 3 fatty acids in the cell membranes.

## Methods

### IRB and obtaining informed consent

The Marshall University Institutional Review Board reviewed and approved the protocol for this study under a Federal Wide Assurance (FWA #00002704) with the Office of Human Research Protections. The purpose and possible risks or benefits were explained to all research participants prior to obtaining signed informed consent from all participants. Blood was obtained at the same time as blood was being obtained for routine clinical tests.

### Subject population

All patients presenting with early stage chronic lymphocytic leukemia were invited to participate in the pilot trial. Patients expressing interest in participating in the trial were then given full details of the study and informed, written consent was obtained.

### Experimental design

The experimental design is shown in Table 1. After obtaining informed consent, two 7 ml tubes of EDTA anticoagulated blood were obtained (time 0) as a baseline and at each subsequent monthly visit. Fatty acid compositions results up to consumption of 9 capsules per day for one month are reported in this paper. Results are from 11 patients at baseline, the same 11 patients after consuming 3 capsules per day for one month, 9 of those patients after consuming 6 capsules per day for one month and 3 of the same patients after consuming 9 capsules per day for one month.

**Table 1:** Experimental design

Time 0	Month 1	Month 2	Month 3	Month 4
Obtain consent, base blood, No n-3	Second baseline Start n-3 at 3 caps/day,	Draw 3 caps specimen Increase to 6 caps/day,	Draw 6 caps specimen Increase to 9 caps/day,	Draw 9 caps specimen

### Description of omega 3 supplement

The supplement was a commercially available (Res-Q, N3 Oceanic, Palm, PA) fish oil concentrate prepared by molecular distillation. It is free from pollutants, PCB's, pesticides and mercury. It is available in capsule or liquid form. Each capsule contained about 400 mg of eicosapentaenoic acid (EPA), 300 mg of docosapentaenoic acid (DHA) and 100-150 mg of other omega 3 fatty acids. Thus, consumption of 3, 6, or 9 capsules/day yielded 2.1 g, 4.2 g, or 6.3 g/day of EPA+DHA, respectively. These amounts are all below the maximum tolerated dose of 13.1 g/day EPA+DHA reported by Burns et al. [22]. Each patient started with a dose of 3 capsules per day (1/meal). Dosage was increased to 6 capsules and then to 9 capsules per day (up to 3/meal) at monthly intervals. Potential dose limiting events were diarrhea, stomach upset or dislike of taking the capsules.

### Specimen handling

An EDTA anticoagulated blood specimen (7 ml) was obtained from the participant at each regular doctor visit and was placed on ice until retrieved by laboratory personnel (within 2-hours of collection). The sample was centrifuged at 400 g for 10 minutes and about 2 ml of plasma was removed, divided to 0.5 ml aliquots and stored at -80°C for future assay. The buffy coat, some plasma and adjacent red blood cells were removed. A Ficoll-Paque (GE Healthcare, Piscataway, NJ) gradient was used to isolate WBCs, according to manufacturer's protocol. WBCs were washed with a glucose containing balanced salt solution, counted and divided into 5 million cell aliquots. Following Ficoll-Paque separation, greater than 95% of cells were lymphocytes.

### White and red blood cell fatty acid composition

The fatty acid compositions of WBCs or RBCs were assessed using gas chromatography, according to our routine techniques, prior to initiation of the omega 3 supplement (baseline, time 0) and after consumption of each dose of the supplement for 1 month. Two hundred microliters of packed red blood cells or 10^6 ^white blood cells were homogenized in distilled water with 0.1% BHT to prevent fatty acid oxidation. Lipids were extracted with chloroform/methanol, then methylated. Methylated lipids were separated and identified using gas chromatography as previously published [23]. Fatty acid methyl ester standards (Nu-Chek-Prep, Elysian, MN) were used for peak identification. The individual fatty acid methyl esters were reported as the percent of the ten analyzed methylated fatty acids (area under the curve). We are especially interested in total omega 6 fatty acids, linoleic acid (LA), gamma linolenic acid, 20:3n-6, and arachidonic acid (AA), and total omega 3 fatty acids, α linolenic acid, EPA, docosapentaenoic acid (DPA) and DHA.

### Statistical Analyses

Prism^© ^software (Graphpad, Inc., La Jolla, CA) was used for statistical analyses by linear regression, non-linear regression, or repeated measures two way analyses of variance (2-way ANOVA) followed by Bonferroni posttests as detailed in the results. Prism^© ^was used for preparation of graphs.

## Competing interests

The authors declare that they have no competing interests.

## Authors' contributions

TRW and AJS performed patient sample handling, processing and GC analyses. OFB was the oncologist and patient physician. OFB and WEH developed the study together. WEH obtained grant funding and wrote the manuscript. All authors have read and approved this manuscript.
